# A *De Novo* Nonsense Mutation in *MAGEL2* in a Patient Initially Diagnosed as Opitz-C: Similarities Between Schaaf-Yang and Opitz-C Syndromes

**DOI:** 10.1038/srep44138

**Published:** 2017-03-10

**Authors:** Roser Urreizti, Anna Maria Cueto-Gonzalez, Héctor Franco-Valls, Sílvia Mort-Farre, Neus Roca-Ayats, Julia Ponomarenko, Luca Cozzuto, Carlos Company, Mattia Bosio, Stephan Ossowski, Magda Montfort, Jochen Hecht, Eduardo F. Tizzano, Bru Cormand, Lluïsa Vilageliu, John M. Opitz, Giovanni Neri, Daniel Grinberg, Susana Balcells

**Affiliations:** 1 Department of Genetics, Microbiology and Statistics, Faculty of Biology, University of Barcelona, IBUB, IRSJD, Barcelona, Spain; 2 CIBERER, Barcelona, Spain; 3 Department of Clinical and Molecular Genetics and Rare Diseases Unit, Hospital Vall d’Hebron, Barcelona, Spain; 4 Centre for Genomic Regulation (CRG), The Barcelona Institute of Science and Technology, and Universitat Pompeu Fabra (UPF), Barcelona, Spain; 5 Pediatrics Medical Genetics, University of Utah School of Medicine, Salt Lake City, Utah, USA; 6 Istituto di Medicina Genomica, Università Cattolica Sacro Cuore, Policlínico A Gemelli, Rome, Italy

## Abstract

Opitz trigonocephaly C syndrome (OTCS) is a rare genetic disorder characterized by craniofacial anomalies, variable intellectual and psychomotor disability, and variable cardiac defects with a high mortality rate. Different patterns of inheritance and genetic heterogeneity are known in this syndrome. Whole exome and genome sequencing of a 19-year-old girl (P7), initially diagnosed with OTCS, revealed a *de novo* nonsense mutation, p.Q638*, in the *MAGEL2* gene. *MAGEL2* is an imprinted, maternally silenced, gene located at 15q11-13, within the Prader-Willi region. Patient P7 carried the mutation in the paternal chromosome. Recently, mutations in *MAGEL2* have been described in Schaaf-Yang syndrome (SHFYNG) and in severe arthrogryposis. Patient P7 bears resemblances with SHFYNG cases but has other findings not described in this syndrome and common in OTCS. We sequenced *MAGEL2* in nine additional OTCS patients and no mutations were found. This study provides the first clear molecular genetic basis for an OTCS case, indicates that there is overlap between OTCS and SHFYNG syndromes, and confirms that OTCS is genetically heterogeneous. Genes encoding *MAGEL2* partners, either in the retrograde transport or in the ubiquitination-deubiquitination complexes, are promising candidates as OTCS disease-causing genes.

Opitz C syndrome (or Opitz-trigonocephaly, OTCS; MIM #211750) is a rare, severe and heterogeneous disorder with some 60 cases described worldwide^1^. Manifestations in this syndrome affect all body systems, are highly variable among different patients, and comprise psychomotor delay, trigonocephaly and a characteristic combination of facial dysmorphisms including retrognathia, upslanted palpebral fissures, and epicanthic folds. Short neck, joint contractures, cryptorchidism, congenital heart malformations and pancreatic and renal involvement may also be present. Commonly, OTCS patients are hypotonic, have arthrogryposis and seizures^2^. The syndrome has a high infant mortality rate and around 50% of patients die during the first year of life mostly due to respiratory failure and cardiovascular malformations^2^. OTCS is similar to other syndromes, mainly to Bohring-Opitz syndrome (BOS or C-like syndrome; MIM #605039), a more severe disorder^3^. The molecular bases of OTCS are still unknown (although *CD96* has been proposed as an OTCS gene^3^, its implication has been recently questioned)^4^,^5^. Likewise, the pattern of inheritance is unclear, with many sporadic cases suggestive of a dominant inheritance and some cases that point to autosomal recessive inheritance because of affected sibs or parental consanguinity. Genetic heterogeneity and clinical variability should be considered in OTCS as in other developmental delay and intellectual disability syndromes.

We previously performed whole exome sequencing (WES) in 5 OTCS patients with normal *CD96* and *ASXL1* genes^5^. Here we present the results of one of them, a 19 year-old Spanish woman, in whom we found a *de novo* nonsense mutation in *MAGEL2*. Whole genome sequencing (WGS), performed in parallel, gave a concordant result after analyzing the exonic data. The *MAGEL2* gene maps to the imprinted Prader-Willi Critical Region (PWCR) on chromosome 15. Several *de novo* truncating mutations, similar to the one described here, have been reported previously in patients with Schaaf-Yang syndrome (SHFYNG, MIM #615547)^6^,^7^, a condition with some resemblances to Prader-Willi syndrome, and in two patients with a distinct severe arthrogryposis phenotype^8^. Very recently, Fountain *et al*.^9^ described 18 additional SHFYNG patients with similar mutations.

Here we present a detailed phenotypic description of this female patient, originally diagnosed as OTCS, and we describe her phenotype in light of the main features of SHFYNG.

## Results

### Clinical Report

**Figure 1:**
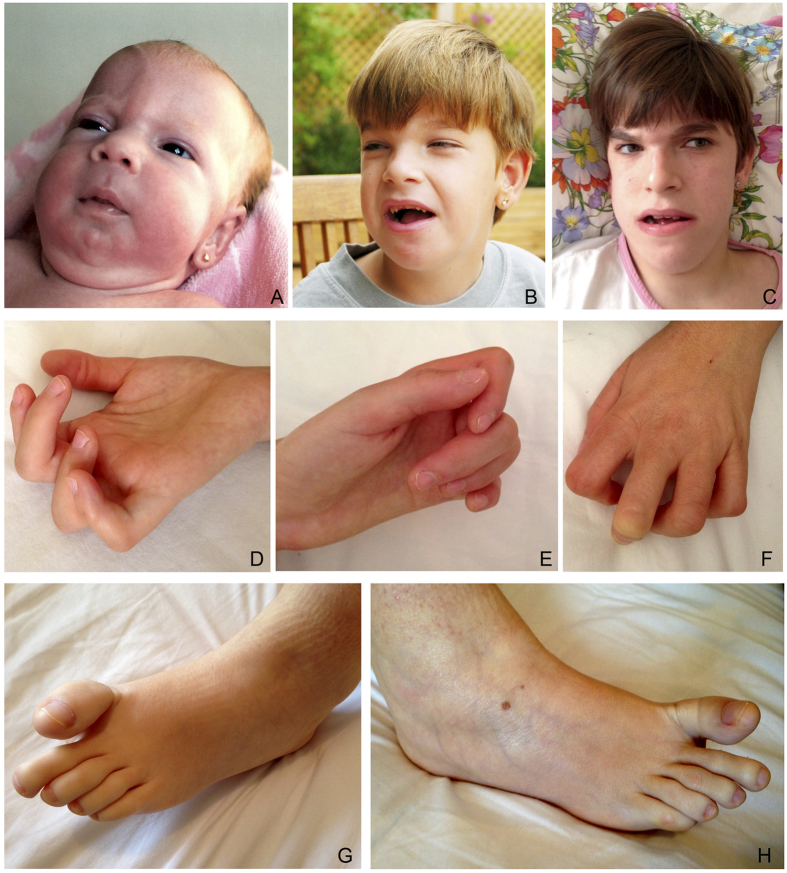
Facial, hand and foot phenotypes of patient P7. (**A**) Age 1 month (trigonocephaly is appreciated); (**B**) 8 years (tooth malposition is appreciated). (**C**–**H**) Age 19 years.

The patient, a 19-year-old woman, is the first and only daughter of a non-consanguineous couple. She was born at full-term by caesarean section with a birth weight of 2.6 kg (34th centile), length of 48 cm (49th centile) and head circumference of 34 cm (61st centile). She had neonatal respiratory depression (Apgar scores 1/5), hypotonia, contractures of fingers and toes and, less evident, bilateral clubfoot. At 1 month she had a cardiopulmonary arrest and bradycardia. In the following months trigonocephaly due to premature metopic suture fusion was noted (Fig. 1A) and at age 2y she was treated neurosurgically. She has had feeding difficulties and oropharyngeal dysphagia and currently eats soft food and liquids, but nothing solid.

There was severe delay in achieving motor milestones and she started to walk with support around age 11y. She has severe intellectual disability with no language, as well as constant sleep disturbances such as long periods of insomnia (up to 72 hours) and difficulties initiating or maintaining sleep. Temperature instability, profuse sweating and excessive salivation occur frequently. Age at menarche was 14y. At 18y she developed sleep apnea and episodic hyperventilation similar to the pattern described in Pitt-Hopkins syndrome. She has suffered multiple infections during her life.

At present (age 19y) her stature, weight and head circumference are below the 3rd centile, and she shows frontal cowlick, strabismus, short nose, slightly anteverted nares, macrostomia, thick palatal and alveolar ridges, teeth malposition (Fig. 1B and C), wide-spaced nipples, hypoplasia of labia majora with prominent clitoris, mild limitation of elbow extension, hands with abnormal palmar creases, hand and feet camptodactyly (Fig. 1D–H) and mild webbing, asymmetric thorax and marked lordosis.

The patient was tentatively diagnosed at 2 years of age as affected with OTCS, confirmed by one of us (JMO) at an Opitz C Syndrome Parent Support Group and Scientific meeting, held in Chicago, Illinois, USA, in 1998.

### Complementary Analyses

Complementary tests and their results (in parentheses) are as follows: karyotype (46, XX); metabolic screening (normal); echocardiogram (normal); cranial CT scan (2y: trigonocephaly); gynaecologic ultrasound (8y: infantile uterus and normal ovaries) cerebral MRI (10y and 13y: thin corpus callosum, inferior vermis hypoplasia, mild brachycephaly, hypophysis of normal size); methylation study of the SNRPN locus within the Prader-Willi region by M-PCR (normal); FISH of the centromeric region of chromosome 12 (normal, Pallister-Killian Syndrome ruled out); sequencing of *TCF4* (no pathogenic mutations, ruling out Pitt-Hopkins syndrome); SNP array (250,000 SNPs, normal).

### Exome and Genome Sequence Analysis

On average, the mean WES coverage for the P7 trio was of 59.1, and 93.2% of the target region was covered by at least 10 reads (C10). See Table S1 for further details.

The main WES result was a *de novo* heterozygous mutation in the *MAGEL2* gene, among some variants of unknown significance in other genes (reported in Table S2). This result was confirmed by an independent WGS.

**Figure 2:**
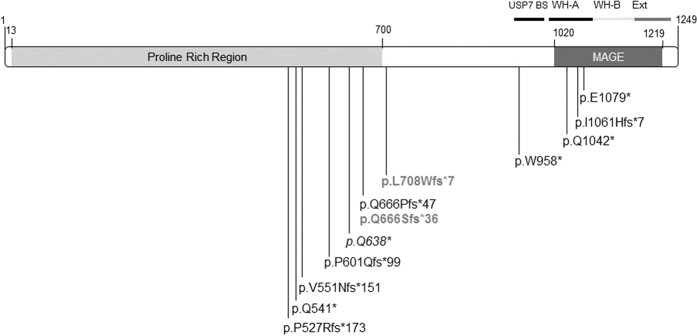
Graphical representation of all the *MAGEL2* pathological mutations reported so far^6^,^7^,^8^,^9^. WH-A and B: Winged-Helix A and B (residues 1013-1097 & 1098-1184); Ext: Winged-Helix B Extension (residues 1185-1227); USP7 BS (USP7 Binding Site, residues 949-1004). In italics, mutation p.Q638* found in the OTCS patient described here and in a SHFYNG patient^9^. Bold and grey represent mutations found in severe arthrogryposis patients^8^,^9^.

The *MAGEL2* mutation consists of a c.1912C > T transition, which leads to the substitution of a glutamine (Q) residue by a STOP codon (p.Q638*) (Fig. 2). Since *MAGEL2* is imprinted and maternally silenced, we experimentally confirmed that the change was present on the paternal chromosome.

### *MAGEL2* Analysis in a Cohort of 9 OTCS Patients

The *MAGEL2* coding region plus 340 bp of the 5′-UTR and 142 bp of the 3′-UTR were Sanger sequenced in 9 other patients diagnosed as OTCS. Patient P2 bore the missense mutation p.A632T [c.1894G > A; ExAC MAF: 0.00003337 (1/29966 individuals)] and patient P12a presented a polymorphic 21-bp in-frame deletion (rs774629250, MAF: 0.0002). Both mutations were present in a heterozygous state and inherited from the respective mothers and, thus, are in the allele predicted to be silenced. We did not find any putatively pathologic mutation in these 9 patients.

## Discussion

In our study, by means of whole-exome and genome sequencing, we have found a *de novo* truncating mutation in the maternally-silenced *MAGEL2* gene in a patient clinically diagnosed as Opitz C. The mutation occurred on the paternal chromosome. No other *MAGEL2* pathogenic mutations were found in any of the remaining 9 OTCS patients that we were able to test. Given the uncertain role of *CD96* in OTCS, as mentioned above, *MAGEL2* might be the first gene clearly associated with Opitz C syndrome. The fact that we did not find any other *MAGEL2* mutation in 9 additional OTCS patients is an indication of genetic heterogeneity of this syndrome.

Truncating mutations in the *MAGEL2* gene were identified previously in two distinct conditions, the Schaaf-Yang syndrome, a relatively mild condition with some resemblance to the Prader-Willi syndrome (see ref. 10 for further comparisons), and in patients with a severe form of arthrogryposis with reduced fetal movement and perinatal death^8^. Recently, Fountain *et al*.^9^, described a large series of SHFYNG patients, all with a truncating *MAGEL2* mutation on the paternal chromosome. Interestingly, they reported three familial cases in which the mutation was inherited from the father. The cosegregation of the mutations and phenotypes in these pedigrees further confirm the pathogenicity of *MAGEL2* truncation.

The patient described here (P7) bears a *de novo* c.1912C > T (p.Q638*) mutation, identical to the mutation present in patient 4 of Fountain *et al*.^9^. In fact, our patient P7 bears good phenotypic resemblance with SHFYNG. She presented with developmental delay, which is observed among all surviving patients with *MAGEL2* mutations, neonatal hypotonia, feeding problems in infancy and arthrogryposis or joint contractures, all traits common to the majority of the patients with *MAGEL2* mutations. Autism spectrum disorder (ASD) -common in *MAGEL2*-mutated patients- could not be evaluated due to her severe intellectual disability. In spite of the age difference, our patient and patient 4 of Fountain *et al*.^9^, with the same *MAGEL2* mutation, share developmental and intellectual disability, feeding problems, neonatal hypotonia, contractures, minor facial anomalies, small hands, sleep apnea and temperature instability. Patient P7 is similar in age and sex to patient 18 of Fountain *et al*.^9^. They are concordant for several features including female hypogenitalism, but discordant with respect to behaviour, sleep apnea, temperature instability and seizures. Worthy of note are a few features present in our patient and not described in the other patients with *MAGEL2* mutations. These include insomnia and sleep difficulties, also observed in *Magel2* null mice^10^, thick palatal and alveolar ridges, weak cry, tooth malposition, trigonocephaly, excessive salivation, episodic hyperventilation and multiple dislocations, many of which are characteristic of OTCS patients.

Despite the high clinical variability observed among patients with mutations in *MAGEL2*, all bear truncating mutations that lead to the formation of a shorter version of the protein lacking the MAGE homology domain (MHD) (Fig. 2). So far, a total of 12 different mutations has been reported. Notably, they cluster in the middle and 3′ portion of the gene, between codons 541 and 1079, of the 1249 of the full *MAGEL2* coding region. There are some recurrent mutations, suggesting the existence of hotspots. It is interesting that one of these hotspots gives rise to two different mutations (c.1996dupC and c.1996delC). One was reported in 12 patients from 8 unrelated families and another in 4 patients from two families. The phenotype associated with the latter is a severe neonatally lethal arthrogryposis, while the former was never associated with this phenotype. This spectrum of phenotypes, with different natural histories, might be gathered under the genetic umbrella of MAGELopathies, in a similar way to RASopathies^11^.

*MAGEL2* belongs to the MAGE (melanoma antigen) domain containing family of proteins^12^, and is known to bind and to enhance the activity of the TRIM27 E3 RING ubiquitin ligase. The MAGEL2-TRIM27 complex, which includes and is regulated by USP7, appears to play a key role in the retrograde transport from the endosome to the trans-Golgi network, an important cellular process which facilitates the recycling of a variety of proteins. Other critical components of this retrograde transport are the three members of the retromer complex (VPS26, VPS29, and VPS35) and the WASH regulatory complex (SHRC), which consists of at least five core factors: CCDC53, FAM21, SWIP, Strumpellin, and WASH^13^,^14^. Interestingly, mutations in different members of the retrograde transport lead to diseases characterized by intellectual disability and developmental delay (Table 1). Mutations in the USP7 are associated with a syndromic form of intellectual disability including features of ASD, hypotonia and seizures^15^. In addition, mutations in SWIP (*KIAA1033*) and strumpellin (*KIAA0196*) were found associated with intellectual disability. In particular, mutations in SWIP have been identified as responsible for a nonsyndromic, autosomal recessive form of intellectual disability with short stature in one family^16^, while strumpellin was associated with two different diseases. On one hand, mutations that lead to the loss of the C-term of the protein were associated with the Ritscher-Schinzel syndrome (RTSC1, MIM#220210), an autosomal recessive condition characterized by intellectual disability, craniofacial abnormalities, congenital heart defects and cerebellar brain malformations^17^. On the other hand, missense mutations were associated with spastic paraplegia 8. In addition, other partners of the WASH complex have been associated with Parkinson disease (PD)^18^, Alzheimer disease^19^ and ASD^20^.

When looking at the photographs of SHFYNG patients^9^ and those of OTCS patients^5^, there are some obvious resemblances. The clinical overlap between these two syndromes suggests that some of the patients initially diagnosed as OTCS may be, in fact, SHFYNG patients and thus, *MAGEL2* should be assessed in OTCS patients. We found only one out of ten OTCS patients with a truncating mutation in *MAGEL2*, but it is likely that other OTCS patients might bear mutations in this gene and we strongly recommend that it should be sequenced in all of them. *MAGEL2* was not well captured in many of the exome assays and, in particular, in capture kits based on the 37 build of the genome the 5′ part of the gene is lacking. For example, Soden *et al*.^7^, investigating neurodevelopmental disorders, mentioned that they found mutations by WGS that were not observed in exome analyses. On the other hand, for *MAGEL*2-negative OTCS patients, the causal genes could be among those coding for other members of the retrograde transport complex or, more specifically, of the ubiquitination and deubiquitination complex. TRIM27 and USP7 form one such ubiquitination-deubiquitination complex^21^. In this regard, USP7 has been recently shown to regulate *ASXL1*^22^, whose mutations are the cause of 50–70% of the cases of Bohring-Opitz syndrome^5^,^23^,^24^,^25^. These functional clues will surely help pinpoint the remaining OTCS causal genes in ongoing whole exome and genome sequencing studies.

## Materials and Methods

### Patients and Samples

The Spanish patient with the *MAGEL2* mutation is P7 in Urreizti *et al*.^7^, together with the four other patients subjected to WES (P1, P2, P6 and P8) and two additional patients (P10a and P11). Three additional patients from three independent families (P12a, P13 and P14) were recruited from Malta, Australia and Spain, respectively, and diagnosed by one of us (either JMO or GN). Written informed consent was obtained from all patients, including a specific informed consent to publish images for patient P7. All methods were carried out in accordance with relevant guidelines and regulations and the Bioethics Committee of the University of Barcelona approved the protocols.

### Exome Sequencing and Filtering

Genomic DNA from patient P7 and her parents was sequenced in the National Center of Genomic Analysis (CNAG; Barcelona, Spain) using the Illumina HiSeq-2000 platform. Exome capture was performed with Nimblegen SeqCap 64 Mb v3 (Roche; Mannheim; Germany). The samples where sequenced at a coverage of 50x. The data were analysed as described elsewhere^26^ (see Supplementary Methods).

### Validation by PCR Amplification and Sanger Sequencing

A total of 75 variants was selected for validation. A primer pair for each fragment containing the position of the putative change was designed using Primer 3^27^ (primer sequences and PCR conditions are available on demand). PCR reactions, purification and sequencing were performed as previously described^5^.

### Genome Sequencing

The whole genomic DNA from the patient and her parents has been sequenced at the CRG Genomics unit. Libraries were prepared using the NEBNext Ultra DNA libray prep kit for Illumina. Sequencing was performed on an Illumina HiSeq2500 instrument, using paired-end 125 bp reads, at the coverage of 36–42x (see Supplementary Methods).

### Phasing the *de novo* Mutation in *MAGEL2*

To assess the phase of the mutation a methylation-sensitive digestion was performed, as in Schaaf *et al*.^6^ with some modifications (see Supplementary Methods).

### PCR Amplification and Sanger Sequencing of the *MAGEL2* Gene

A region of 4.2 kb including the *MAGEL2* coding region, 304 bp of the 5′-UTR and 143 of the 3′-UTR (GRCh38 ENSG00000254585) was amplified in 9 overlapping fragments prior to Sanger sequencing. Primer pairs and PCR conditions are summarized in Table S3.

## Additional Information

**How to cite this article**: Urreizti, R. *et al*. A *De Novo* Nonsense Mutation in *MAGEL2* in a Patient Initially Diagnosed as Opitz-C: Similarities Between Schaaf-Yang and Opitz-C Syndromes. *Sci. Rep.* **7**, 44138;doi: 10.1038/srep44138 (2017).

**Publisher's note:** Springer Nature remains neutral with regard to jurisdictional claims in published maps and institutional affiliations.

## Supplementary Material

Supplementary Information

## Figures and Tables

**Table 1:** Clinical findings in patients with mutations in genes involved in endosome to trans-Golgi network retrograde transport.

	MAGEL2	USP7	KIAA1033 (SWIP)	KIAA0196 (Strumpellin)
Syndrome	Schaaf-Yang	Mental retardation	Mental retardation 43	Ritscher-Schinzel syndrome 1
	Sev. arthrogryposis			
	Opitz C			
Patients	29	7	7	10
Families	22	7	1	9 [1]
Gender	17M; 12F	5M; 2F	2M; 5F	6M; 4F
Mutation type	all truncating	6 del; 1 nonsense	missense	splicing (truncating)
Inheritance	AD	AD	AR	AR
**Development and Behaviour**				
DD/ID	21/21	7/7	7/7	10/10
Speech articulation defects/Apraxia of speech	3/5	4/7	7/7	NR
Characteristic behavior (temper tantrums, violent outbursts, oppositional behavior, etc.)	11/15	4/7	NR	NR
Autism spectrum disorder	10/13	5/6	NR	NR
Sleep apnea and sleep disturbances	11/17	1/7	NR	NR
**Physical Characteristics**				
Hypotonia	14/14	4/7	NR	NR
Feeding problems in infancy, with need for special feeding technique	21/23	NR	NR	NR
Short stature	13/21	NR	7/7	NR
CNS structural anomaly (Dandy-Walker malformation, Hypoplastic cerebellar vermis, others)	NR	2/7	NR	9/10
Skin picking	9/16	NR	NR	NR
Dysmorphic facial features [2]	22/27	1/7	NR	10/10
Eye abnormalities (exotropia, myopia, strabismus and coloboma)	15/19	2/7	NR	10/10 [3]
Craniosynostosis	1/1[4]	1/7	NR	10/10
Scoliosis, kyphosis or lordosis	9/19	NR	NR	NR
Contractures	24/29	NR	NR	NR
Small hands	14/19	1/7	NR	NR
Hand malformation (narrow hands, brachidactyly, clinodactyly)	1/5	2/7	NR	6/10
Hypogonadism	11/15M; 2/2F	4/5	NR	0/10

[1] Two patients are 3rd cousins. [2] Including downslanting palpebral fisures, low set ears, malformation of the philtrum, nasal structure and palpebral fissure, micro, retro or prognathia. [3] All the patients presented with coloboma. [4] This condition has not been specifically reported in previous publications but some degree of craniosynostosis could be appreciated in some of the patient’s pictures at ref. 9. NR = Not Reported; DD/ID = Developmental delay, Intellectual disability; AD = Autosomal dominant; AR = Autosomal recessive.
