# Supplementary material for: A De Novo Nonsense Mutation in MAGEL2 in a Patient Initially Diagnosed as Opitz-C: Similarities Between Schaaf-Yang and Opitz-C Syndromes
Source: Sci Rep. 2017 Mar 10;7:44138. doi: 10.1038/srep44138 (PMC5345063; doi:10.1038/srep44138)
Supplement: Supplementary Information [file srep44138-s1.pdf]

A *DE NOVO* NONSENSE MUTATION IN *MAGEL2* IN A PATIENT INITIALLY DIAGNOSED AS OPITZ-C: SIMILARITIES BETWEEN SCHAAF-YANG AND OPITZ-C SYNDROMES

Roser Urreizti, PhD, Anna Maria Cueto-Gonzalez, MD, Héctor Franco-Valls, BSc, Sílvia Mort-Farre, MSc, Neus Roca-Ayats, MSc, Julia Ponomarenko, PhD, Luca Cozzuto, PhD, Carlos Company, MSc, Mattia Bosio, PhD, Stephan Ossowski, PhD, Magda Montfort, PhD, Jochen Hecht, PhD, Eduardo F. Tizzano, MD, PhD, Bru Cormand, PhD, Lluïsa Vilageliu, PhD, John M. Opitz, MD, Giovanni Neri, MD, Daniel Grinberg D, PhD, Susana Balcells, PhD

SUPLEMENTARY INFORMATION

Suplementary Methods:

Exome Sequencing and Filtering

The results were filtered under *de novo* dominance and recessive hypotheses. Variants with a MAF above 0.001 (under the dominant) and above 0.01 (for recessive) in the common population (according to ExAC and 1000 genomes) were excluded. Variants in genes included in selected databases [The Development Disorder Genotype - Phenotype Database (DDG2P)][1,2] and covered by at least 10 reads were prioritized for validation (it should be noted that those who carried out the original DECIPHER analysis and collection of the data bear no responsibility for the further analysis or interpretation of it). In parallel, variant effects were classified as high, moderate or low according to SnpEff [3] and mutations with a high putative effect and at least 10 reads were also prioritized for validation by Sanger sequencing.

Whole Genome Sequencing

Reads were aligned to the human genome (hg19) using BWA mem (v.0.7.10)[4]. GATK (v.3.2.2)[5] was used for local re-alignment and calling of SNVs that were annotated with two pipelines, EDiVa (Exome-seq based Disease Variant analysis platform) [http://www.ediva.crg.eu/] and myPhenoDB [https://phenodb.org/]. EDiVa annotates variations and affected genes using data from a number of publicly available databases (dbSNP, 1000Genomes, OMIM, and other), considering different inheritance models in the family trio. The software also evaluates the effects of a given variation on the coding protein using a number of predictive tools (SIFT, Polyphen2, Condel, and other).

In the result, EDiVa identified two compound and two *de novo* mutations affecting three genes, among which was the *de novo* nonsense mutation affecting the *MAGEL2* gene. PhenoDB detected 93 *de novo* autosomal dominant SNVs, applying a cut off on the CCDS intolerance percentile of 10% and retaining public SNPs with frequency smaller than 0.005. OMIM data were available for four genes in which *de novo* SNVs were identified. The nonsense mutation in *MAGEL2* was detected and classified as “pathogenic”, while the other three mutations were classified as “likely pathogenic” and of “uncertain significance”.

Phasing the *de novo* Mutation in *MAGEL2*

One hundred ng of the patient and parent’s gDNA was digested with the methylation-sensitive enzyme *Sma*I (Fermentas, Thermo Fischer Scientific, Waltham, MA, EUA). The PCR products were purified with the MultiScreenTM

Vacuum Manifold 96-well plate (Merck Millipore, Bellerica, MA, USA) following the manufacturer's instructions and quantified using a NanoDrop ND-1000 Spectrophotometer (Nanodrop Technologies Inc., Wilmington, DE, USA). A 2.28 kb region including the mutation and methylation sites was amplified using primers MAGEL2-LR-F and MAGEL2-LR-R (Table S1) under the following conditions: 0.2 μM of each dNTP, 0.4 μM of each primer, 5% DMSO, 2.5 mM Mg^{2+} and 0.7 u of GoTaq Flexy (Promega, Madison, WI, USA) in the presence of 100 ng of *Sma*I digested gDNA. The reaction was performed as follows: initial denaturation step of 5 minutes at 95°C, 35 cycles of 30 seconds at 95°C, 30 seconds at 57°C and 30 seconds at 72°C, followed by a final extension of 5 minutes at 72°C.

Supplementary References:

1. Firth HV, Richards SM, Bevan AP, et al. DECIPHER: Database of Chromosomal Imbalance and Phenotype in Humans Using Ensembl Resources. *Am J Hum Genet* 2009;84:524-533.
2. Samocha KE, Robinson EB, Sanders SJ, et al. A framework for the interpretation of de novo mutation in human disease. *Nat Genet* 2014;46:944-950.
3. Cingolani P, Platts A, Wang le L, et al. A program for annotating and predicting the effects of single nucleotide polymorphisms, SnpEff: SNPs in the genome of Drosophila melanogaster strain w1118; iso-2; iso-3. *Fly (Austin)* 2012;6:80-92.
4. Li H, Durbin R. Fast and accurate long-read alignment with Burrows-Wheeler transform. *Bioinformatics* 2010;26:589-595.
5. McKenna A, Hanna M, Banks E, et al. The Genome Analysis Toolkit: a MapReduce framework for analyzing next-generation DNA sequencing data. *Genome Res* 2010;20:1297-1303.

Supplemantary Tables:

Table S1. WES coverage in P7 and parents

	Total Kb	C10	Mean cov	Median cov
**P7**	588756.2	93.3	59.2	53
**P7p**	625121.2	93.4	62.9	56
**P7m**	548426.0	92.9	55.2	49
**Average**	**587434.5**	**93.2**	**59.1**	**52.7**

Table S2. Main exome findings in patient P7

Gene	Mutation	Position	Inheritance	SIFT	PolyPhen	Constrained[1]	Comments
MAGEL2	p.Q638*	15:23890978 (G>A)	AD (de novo, paternal chr.)	-	-	LoF	Schaaf-Yang syndrome (AD)
CLEC12B	p.E105K	12:10167244 (G>A)	AR (paternal)	T	B	no	MAF: 0.00005784
CLEC12B	p.S210F	12:10168275 (C/T)	AR (maternal)	D	D	no	MAF: 0.000008240
ANKK1	p.R122H	11:113264382 (G>A)	AR (paternal/maternal)	T	B	no	7 homozygotes in ExAc (MAF: 0.009059)

1. According to ExAC.

T: Tolerated; B: Benign; D: Deleterious

Table S3. Primers for the amplification of *MAGEL2*

Name	Sequence	Tm	Mg	Steps	Fragment size
MAGEL2-frg1a-F	TCTGACTGGTCTGCATTTGG	60	1.5	2	377
MAGEL2-frg1a-R	GGCTATAGACAGGCGGCTTCG				
MAGEL2-frg1b-F	AGCTAAGTAAGAATCTGGGTG	58	1.5	2	482
MAGEL2-frg1b-R	AGGAGGATGGGCCATTGGG				
MAGEL2-frg2a-F	ATGGTGCATCCTCCACCTCC	68	1.5	2	459
MAGEL2-frg2a-R	CTGGACCATCGGTGCTCCC				
MAGEL2-frg2b-F	ACTCCGGGAGTCCTGATGGT	60	1.5	2	305
MAGEL2-frg2b-R	ATAACTTGAGACTGGATTTGCAG				
MAGEL2-frg3-F	CCTCCAGCTTCAGGAGCAC	63	1.5	3	747
MAGEL2-frg3-R	GGTAGCAGGTGGGGCCGTA				
MAGEL2-frg4-F	CCACCCCCACCTCCACTG	61	2	2	701
MAGEL2-frg4-R	ATCATGCGGTCTTTTGAAGG				
MAGEL2-frg5-F	AGAATGCAGGGCCTCTTCTA	60	1.5	2	722
MAGEL2-frg5-R	CTTCCCAGCCACTCAGGAT				
MAGEL2-frg6-F	AGGCCCTGGGAGAATCTAAA	60	1.5	2	756

MAGEL2-frg6-R	CCTGACAAACACTTCGGTGA				
MAGEL2-frg7-F	AGTTTGGCCTTCTGATGGTG	60	1.5	2	567
MAGEL2-frg7-R	TTTGGCAGATACGAAACCAA				
MAGEL2-LR-F	ACTCACTTCCTATTCAGCATTCAGC	58	2.5	3	2284
MAGEL2-LR-R	CTGATGGAGTCATCAATGATTTAGC				
